# Supplementary material for: Trophic degradation predispositions and intensity in a high-flow, silted reservoir
Source: PeerJ. 2020 Jun 22;8:e9374. doi: 10.7717/peerj.9374 (PMC7359821; doi:10.7717/peerj.9374)
Supplement: Supplemental Information 1 [file peerj-08-9374-s001.docx]

**Methodology details**

**Assessment system of the susceptibility to degradation**

According to the assessment system developed by Bajkiewicz-Grabowska (1987, 2010), points from 0 to 3 are awarded to individual features and parameters of the evaluated reservoir and its catchment (Tab. 1, 3). Based on the arithmetic average of the points awarded, the category of reservoir resilience to degradation and the group of catchment susceptibility to the supply of matter to it are determined (Tab. 2, 4).

**Table 1. Resilience assessment to degradation of the reservoir (Bajkiewicz – Grabowska, 1987, 2010).**

| **Parameters** | **Points total** | | | |
| --- | --- | --- | --- | --- |
|  | **0** | **1** | **2** | **3** |
| Average depth [m] | > 10 | 5-10 | 3-5 | < 3 |
| Ratio of reservoir capacity [‘000 m^3^] to length of shoreline [m] | > 5 | 3-5 | 1-3 | < 1 |
| Participation of the water stratification [%] | > 35 | 20-35 | 10-20 | < 10 |
| Ratio of the active sediment layer surface [m^2^] to volume of epilimnion [m^3^] | < 0.10 | 0.10-0.15 | 0.15-0.30 | > 0.30 |
| Intensity of water exchange | > 10 | 5-10 | 1-5 | < 1 |
| Schindler’s ratio* [m^2^ ∙ m^-3^] | < 10 | 10-30 | 30-100 | > 100 |

* Ratio of the total area of the catchment and the reservoir to the volume of reservoir.

**Table 2. Resilience categories to degradation of the reservoir (It was compiled on**

**the basis of Bajkiewicz – Grabowska, 1987, 2010).**

| **Arithmetic** average | Category | [**Description**](https://www.diki.pl/slownik-angielskiego?q=description) |
| --- | --- | --- |
| ≤ 0.8 | I | highly resilience on the impact of the catchment |
| od 0.9 do 1.6 | II | moderately resilience on the impact of the catchment |
| od 1.7 do 2.4 | III | little resilience on the impact of the catchment |
| > 2.4 | IV | practically not resilience on the impact of the catchment |

**Table 3. Susceptibility assessment of catchment for the supply of matter into the reservoir (Bajkiewicz – Grabowska, 1987, 2010).**

| **Parameters** | **Points total** | | | |
| --- | --- | --- | --- | --- |
|  | **0** | **1** | **2** | **3** |
| Ohle's coefficient* | < 10 | 10-40 | 40-150 | > 150 |
| Balance type of lake | - | Exorheic | Endorheic | Flow-through |
| Average slope in the catchment [‰] | < 5 | 5-10 | 10-20 | > 20 |
| Geological structure of the catchment | clayey | sandy - clayey | clayey – sandy | sandy |
| Usage of the catchment | forest, agricultural - forest, pasture - agricultural - forest, pasture-forest | forest - agricultural,  pasture - agricultural | agricultural,  pasture - forest - agricultural with buildings,  forest with buildings | forest - agricultural with buildings,  pasture - agricultural with buildings,  agricultural with buildings |
| Density of river network [km•km^-2^] | < 0.5 | 0.5-1.0 | 1.0-1.5 | > 1.5 |
| Contribution of endorheic areas [%] | > 60 | 45-60 | 20-45 | < 20 |

* Ratio of the total catchment area to the reservoir area.

**Table 4. Susceptibility groups of catchment for the supply of matter into the reservoir (It was compiled on the basis of Bajkiewicz – Grabowska, 1987, 2010).**

| **Arithmetic** average | Group | [**Description**](https://www.diki.pl/slownik-angielskiego?q=description) |
| --- | --- | --- |
| ≤ 1.0 | 1 | practically no matter reaching the reservoir |
| od 1.1 do 1.4 | 2 | low supply of matter to the reservoir |
| od 1.5 do 1.9 | 3 | moderate possibility of matter reaching the reservoir |
| ≥ 2.0 | 4 | large possibility of material delivery to the reservoir |
